# Resistance to 16-Membered Macrolides, Tiamulin and Lincomycin in a Swine Isolate of *Acholeplasma laidlawii*

**DOI:** 10.3390/antibiotics10111415

**Published:** 2021-11-19

**Authors:** María M. Tavío, Ana S. Ramírez, Carlos Poveda, Rubén S. Rosales, Cristina F. Malla, José B. Poveda

**Affiliations:** 1 Microbiología, Facultad de Ciencias de la Salud, Universidad de Las Palmas de Gran Canaria, 35016 Las Palmas, Spain; mariadelmar.tavio@ulpgc.es (M.M.T.); cristina.malla101@alu.ulpgc.es (C.F.M.); 2 Unidad de Epidemiología y Medicina Preventiva, Instituto Universitario de Sanidad Animal y Seguridad Alimentaria (IUSA), Universidad de Las Palmas de Gran Canaria, 35413 Arucas, Spain; carlos.poveda103@gmail.com (C.P.); ruben.rosales@ulpgc.es (R.S.R.); jose.poveda@ulpgc.es (J.B.P.)

**Keywords:** macrolide, tiamulin, lincomycin, antimicrobial resistance, *Acholeplasma laidlawii*

## Abstract

*Acholeplasma* (*A.*) *laidlawii* is an opportunistic pathogen with the ability to disseminate resistance determinants to antibiotics; however, its resistance to macrolides has been less studied. The aim of the present study was to characterize the mechanisms responsible for the resistance to macrolides, tiamulin and lincomycin found in a strain of *A. laidlawii* isolated from a pig with pneumonia. MICs of erythromycin, 15- and 16-membered macrolides, tiamulin and lincomycin were determined by microdilution method with and without reserpine, an inhibitor of ABC efflux pumps and regions of the genome were sequenced. Reserpine only decreased lincomycin MIC but it did not change the MICs of macrolides and tiamulin. The analysis of the DNA sequence of 23S rRNA showed nucleotide substitutions at eight different positions, although none of them were at positions previously related to macrolide resistance. Five mutations were found in the L22 protein, one of them at the stop codon. In addition, two mutations were found in the amino acid sequence of L4. The combination of multiple mutations in the ribosomal proteins L22 and L4 together with substitutions in 23S rRNA DNA sequence was associated with the resistance to macrolides, the pleuromutilin and lincomycin in the studied *A. laidlawii* strain.

## 1. Introduction

*Acholeplasma* (*A*.) *laidlawii*, a species of the class Mollicutes, is universally disseminated, having been first isolated from wastewaters in 1936 [1]. It colonizes animals, plants and insects, and it is a frequent contaminant in cell cultures and vaccine preparations [2,3,4,5]. It is frequently found as commensal of the mucosa in the upper respiratory and in the urogenital tract in animals, although it is able to cause opportunistic infections such as bovine mastitis [6,7,8]. In addition, the inoculation of *A. laidlawii* PG-8 into the teat canal resulted in clinical mastitis and agalactia in goats and sheep [8,9]. Likewise, its intranasal inoculation was associated with the development of arthritis and respiratory distress in lambs [9]. *A. laidlawii* has been also isolated from both normal and pneumonic lungs of pigs [10] and nasal secretion, trachea, and lung of calves [11].

Resistance to fluoroquinolones has been further studied in in vitro selected mutants of *A. laidlawii*, especially in relation to the ability of this species to produce extracellular vesicles carrying target genes of fluoroquinolones in addition to the presence of genes responsible for the development of resistance to beta-lactams in other bacterial species [12,13]. Resistance to macrolides other than erythromycin [14] in clinical isolates has not been described in *Acholeplasma* species, although multiple drug-resistant strains can be easily selected in vitro [15]. In the present work, we characterized the substitutions in DNA encoding 23S-ribosomal RNA and mutations in the ribosomal proteins L22 and L4 in a Macrolide-Lincosamide-Pleuromutilin (MLP)-resistant *A. laidlawii* strain isolated from the nostrils of a pig with pneumonia.

## 2. Results

PCR analysis of nucleotide sequence of 16S rRNA and the 16S-23S rRNA intergenic spacer region confirmed *A. laidlawii* species identification. MICs for 470 wild strain of all the 15- and 16-membered macrolides were ≥32 mg/L and erythromycin and lincomycin MICs were 2048 and 4096 mg/L, respectively, whereas MICs of the 15- and 16-membered macrolides and lincomycin ranged < 0.25–1 mg/L for the type strain PG8, with the exception of erythromycin, which produced a similar value to that observed for the wild strain (2048 mg/L). Reserpine 20 mg/L did not increase the susceptibility of the 470 strain to macrolides, tiamulin and lincomycin (Table 1).

Multiple nucleotide substitutions were found in DNA sequences of the 23S rRNA and the proteins L22 and L4 codifying genes. The analysis of the 23S rRNA DNA sequence showed nucleotide substitutions at eight different positions with respect to *A. laidlawii* PG-8A, shown in the corresponding domains (Table 2) following *Escherichia* (*E.*) *coli* numbering [16]. Furthermore, ten nucleotide substitutions were found in the DNA sequence of L22 (A70T, T84C, T144A, G279A, A312T, G322A, C323G, G325A, A328G, T334A), six of which resulted in amino acid changes (Table 2). In addition, thirteen nucleotide substitutions were found in the DNA sequence of L4 (A67T, T68C, C123A, C171T, G318A, A357T, A360T, A390T, T426C, T468A, G517T, T522A, A540G), although only three of them resulted in amino acid changes in the L4 protein (Table 2).

## 3. Discussion

The family *Acholeplasmataceae* is composed of the genus *Acholeplasma* and the phytoplasmas which have evolved by further degenerative evolution. It has been demonstrated that *A. laidlawii* and phytoplasmas can infect plants in a similar way. Phytoplasmas are characterized by their high genomic plasticity that allows them the acquisition and loss of genes that can result in the carriage of adhesion and virulence factors and the colonization and survival in a broad range of environments and hosts [1]. Within this family, *A. laidlawii* is the most studied species [1] and it is considered to be a hypermutable microorganism [5].

*A. laidlawii* strain 470 was isolated from the nostril exudate of a pig with pneumonia. PCR and sequencing analysis confirmed the species, including the characteristic double band for the intergenic spacer region, as previously described [17,18]. Although the species is more commonly associated with mastitis, previous studies have also isolated it from nasal secretions [11]. Strain 470 was resistant to erythromycin and clarithromycin due to the transition A2057G (*E. coli* numbering) in the nucleotide sequence 2056-GGAAAGAC-2063 of domain V of the 23S rRNA-encoding DNA. The G-to-A transition at position 2057 (*E. coli* numbering) was previously associated with the intrinsic resistance to 14-membered ring macrolides including erythromycin and clarithromycin in *M. pulmonis*, *M. hyopneumoniae*, and *M. flocculare* [19,20]. The same transition A2057G (*E. coli* numbering) was found in 23S rRNA DNA sequences of the type strain *A. laidlawii* PG-8A NC_010163.1 (75178.78004) and in strain 470 associated with erythromycin MICs of 2048 mg/L. In addition, the MIC values for erythromycin described for the type strain PG8 further support the potential role of intrinsic resistance in *A. laidlawii* to this antibiotic. Azithromycin MIC in strain 470 was 4- to 64-fold higher than those described in *M. fermentans* PG28, *M. pulmonis* UAB CTIP and *M. hominis* PG21 which are strains with the substitution A2057G in the 23S rRNA DNA sequence [19], although azithromycin MIC in *M. hominis* PG21 was also described to be 4- to 16-fold higher than in *M. pulmonis* UAB CTIP and *M. fermentans* PG28, respectively [19]. Likewise, strain 470 was resistant to the assayed 16-membered macrolides tylosin and tilmicosin, since MIC values of the above 16-membered macrolides were ≥16 mg/L, as was previously described [21]. Strain 470 was also resistant to the pleuromutilin tiamulin and to lincomycin. Previous reports support the role of ABC transporters in the resistance to antibiotics in mollicutes [13], in this regard ABC-type efflux pump has been involved in macrolide resistance in *M. pneumoniae* [22]. Furthermore, the role of ATP-dependent efflux pumps in resistance to ciprofloxacin in *A. laidlawii* was previously described [23]. The inhibition of ATP-dependent active efflux by the use of reserpine 20 mg/L, an inhibitor de ATP-dependent active efflux in mycoplasmas [24], only decreased lincomycin MIC in strain 470.

The search of mechanisms responsible for the high level of resistance to macrolides and lincomycin in strain 470 revealed eight nucleotide substitutions in the 23S rRNA-encoding DNA, although they were not at positions 2058 and 2059 (*E. coli* numbering), which are strongly associated with resistance to 16-membered macrolides [25], neither in other nucleotides of 2056-GGAAAGAC-2063 (*E. coli* numbering) except the G2057A transition, unlike previous descriptions on 16-membered macrolide-resistant mycoplasmas [25]. There was neither substitution at position 2611 (*E. coli* numbering) in the 23S rRNA-encoding DNA, which has been also associated with a low level of resistance to Macrolides Lincosamides Streptogramins Ketolides (MLSK) [25].

Single amino acid changes in conserved regions of L4 and L22 proteins have been previously described in mycoplasmas resistant to macrolides [25,26]. Mutations in L4 and L22 can modify the macrolide-binding site [27]. In this regard, a previous description found that the suppression of four amino acids between threonine 110 and lysine 115 in L22 of *M. pneumoniae* resulted in the complete loss of telithromycin activity, a semi-synthetic erythromycin derivative [20]. However, the four amino acids occupying the equivalent position in L22 of *A. laidlawii* PG-8A, those between methionine 85 and lysine 90, were not mutated in strain 470. Conversely, ten nucleotide substitutions were identified in L22 of strain 470 and six of them resulted in amino acid changes including non-stop mutation at position 112 that generated a protein with an extreme length and probably with a distortion of the target of macrolides. The mutated L22 protein did not prevent the growth of strain 470, as previously described in a strain of *Bacillus subtilis* in which the inactivation of the gene encoding L22 protein did not avoid cell proliferation [28]. Likewise, two mutations were found in L4. We hypothesize a possible perturbation in the global folding of rRNA during 50S subunit assembly in strain 470 by the interaction of mutated L22 and L4 with rRNA resulting in macrolides, tiamulin and lincomycin resistance. In fact, a genotype consisting of single and double nucleotide substitutions in the domain II of the 23S rRNA DNA together with a single mutation in L22 was described associated with tylosin MICs ≥ 8 mg/L and tilmicosin MIC ≥ 128 mg/L in *M. bovis* [27], corresponding to 702 and 706 nucleotides in domain II and Leu86 in L22 of *A. laidlawii* PG8-A, although changes in these last positions were not identified in strain 470. Furthermore, the presence of Lys at position 90 in L22 (Lys90) in the reference strain *A. laidlawii* PG-8A was not associated with macrolide-lincosamide resistance, unlike the single mutation Arg97Lys in L22 in *M. hominis* associated with the macrolide-lincosamide-streptogramin B (MLS_B_) resistance phenotype [20]. There are multiple descriptions of mutations in L4 and L22 ribosomal proteins that have been associated with macrolide resistance in several *Mycoplasma* species [12,29]. Strain 470 showed five and two mutations in L22 and L4, respectively, which has not been previously described. It is possible that some or all of these mutations were able to influence the functionally active three-dimensional structure of 23S rRNA at multiple sites, as Gregory and Dahlberg (1999) [30] suggested for *E. coli*, impacting macrolides, tiamulin and lincomycin binding and resulting in resistance to them.

To our knowledge, this is the first report on a multi-drug resistant clinical isolate of *A. laidlawii* from a pig carrying multiple mutations in the 50S ribosomal proteins L22 and L4 and nucleotide substitutions in the DNA sequence of 23S rRNA. The combination of multiple mutations in the ribosomal proteins L22 and L4 together with substitutions in 23S rRNA DNA sequence were associated with increased resistance against macrolides; however, these findings must be confirmed by introducing these mutations individually and/or in combination into the susceptible strain of *A. laidlawii*. The challenge of a possible spread of resistant *A. laidlawii* strains helped by their genomic plasticity should not be overlooked.

## 4. Materials and Methods

### 4.1. A. laidlawii Strains

*A. laidlawii* 470 was isolated from the nostrils of a pig with pneumonia from an Italian farm in 2016. Tylosin, tiamulin tilmicosin and tylvalosin were routinely used for the systemic treatment of pneumonia on this farm. Those antibiotics were used alternatively during the production cycle. The field strain was isolated in Friis medium supplemented with horse and porcine sera and without antibiotics as described and filtered and cloned to ensure purity [31,32]. The strain was identified by their morphologic and biochemical properties [33], and the identification was confirmed by PCR by sequencing 16S rRNA, 23S rRNA and the 16S to 23S rRNA intergenic spacer region, as described below. We used *A. laidlawii* strain PG8^T^ as a control strain for MIC determinations, obtained from the collection of microorganisms of Mycoplasma Experience Ltd. (Surrey, UK).

### 4.2. Antimicrobials

The antimicrobial agents used in this study included tilmicosin, clarithromycin, azithromycin, erythromycin and tiamulin from Fluka Analytical (St. Louis, MO, USA), tylosin from Serva (Heidelberg, Germany) and lincomycin, a lincosamide, from Sigma (St. Louis, MO, USA). The stock antibiotic solutions were sterilized by filtration through 0.2 µm pore size membrane filters (Millipore, Madrid, Spain).

### 4.3. Determination of MICs

MICs of erythromycin and clarithromycin, two 14-membered ring macrolides and azithromycin a 15-membered ring macrolide, and MICs of the 16-membered ring macrolides tylosin and tilmicosin, the pleuromutilin tiamulin and the lincosamide lincomycin were determined by using the broth microdilution method, as previously described [34], modified by using Friis medium supplemented with 0.2% phenol red and 1% glucose. Serial twofold dilutions of each antibiotic ranging from 0.003 to 256 mg/L were prepared for 15- and 16-membered macrolides and tiamulin and 0.12 to 4096 mg/L for erythromycin and lincomycin in sterilized microdilution plates. MICs were determined in strains 470 and PG8^T^ at the same time and in the same conditions, as previously described [32,34]. The strains were tested in duplicate on three different days for each agent, with and without reserpine 20 mg/L, which has been proved to be sufficient to inhibit adenosine triphosphate (ATP)-binding cassette (ABC) efflux pumps in mycoplasma strains [34,35].

### 4.4. Polymerase Chain Reaction Amplification and Sequencing

Genomic DNA was extracted from *A. laidlawii* 470 culture using an UltraClean Microbial DNA isolation Kit (MO BIO Laboratories, Carlsbad, CA USA). PCR analysis of the nucleotide sequence of the 16S rRNA and the 16S to 23S rRNA intergenic spacer region for species identification were performed as previously described [36,37]. Likewise, different primers were designed by using as reference the sequences of *A. laidlawii* PG-8A present at GenBank Database and used for the complete amplification of DNA encoding 23S ribosomal RNA NC_010163.1 (75178.78004), as well as DNA sequences of proteins L22 NC_010163.1 (87002.87337) and L4 NC_010163.1 (84911. 85534) of the 50S ribosomal subunit (Table 3). The PCR products were purified using the E.Z.N.A. cycle pure kit, MO BIO Laboratories, California (USA). Finally, purified PCR products were sequenced by an external service (Macrogen, Madrid, Spain). Nucleotides sequences were analyzed using BLAST online service (https://blast.ncbi.nlm.nih.gov/Blast.cgi, accessed on 21 November 2019).

## 5. Conclusions

Mutations in five amino acids were found in L22, one of them in the stop codon that resulted in an extremely long and probably dysfunctional protein, in conjunction with mutations in L4 and substitutions in 23S rRNA DNA sequence were associated with the resistance profile to macrolides, the pleuromutilin and lincomycin in the studied *A. laidlawii* strain 470.

## Figures and Tables

**Table 1:** MICs of macrolides, tiamulin and lincomycin in *Acholeplasma laidlawii* PG8T and in 470 without and with reserpine 20 mg/L.

	MIC (mg/L)	
Antibiotics	*A. laidlawii* PG8^T^	*A. laidlawii* 470
Erythromycin	2048	2048
Erythromycin + Reserpine *	2048	2048
Clarithromycin	0.25	32
Clarithromycin + Reserpine	-	32
Azithromycin	0.125	128
Azithromycin + Reserpine	-	128
Tylosin	0.25	64
Tylosin + Reserpine	-	64
Tilmicosin	0.25	64
Tilmicosin + Reserpine	-	128
Lincomycin	<0.25	>4096
Lincomycin + Reserpine	-	4096
Tiamulin	1.0	128
Tiamulin + Reserpine	-	128

* Reserpine assay for *A. laidlawii* type strain PG8 was performed in order to evaluate the role of efflux pump for erythromycin resistance.

**Table 2:** Nucleotide substitutions in DNA encoding 23S ribosomal-RNA and mutations in the ribosomal proteins L22 and L4 in *Acholeplasma laidlawii* 470 comparing to type strain PG8.

	Nucleotide Changes				Mutations	
Strain	23S Ribosomal RNA Domains ^1^				L4	L22
	I	II	III	V		
470	G320A	T1254G **^2^**	G1376A G1450T G1475A G1579A	T2131A T2133G	A67T T68C Ile23Ser ATC→TCC G517T Ala173Ser GCA→TCA	A70T Ile24Phe ATT→TTT G322A C323G Ala108Arg GCA→AGA G325A Glu109Lys GAA→AAA A328G Arg110Gly AGG→GGG T334A STOP112Lys TAA→AAA

^1^ Nucleotide substitutions following *Escherichia coli* numbering and using the canonical 2° structure with 6 domains [16]. ^2^ This position is in Domain 0 based on a 3D structure with 7 domains [16].

**Table 3:** Primers used in the study for PCR and sequencing.

Gene	Forward Oligonucleotide Sequences (5′-3′)	Reverse Oligonucleotide Sequences (5′-3′)
23S-rRNA	GAACAAAGGGCACACAGTG	CTTGCTATGTAACATAACTCGC
	GATGGCATGCCTTTTGTAG	CAGAGTCACTCGACCAGTG
	GCCATCCTTTAAAGAGTGCG	CAACAGTTTTCTCGCGCGTC
	GTAAACCGACACAGGTGG	CAAACTGCCCACCAGACAC
	CGTGCACTTAGTTTCTAACTTC	CCAGTAAGCTGAATACATCGC
*rplV* ^1^	GAAGCTAAAGCAATTGGAAAAAC	CTCCCTTTCTGCGACAAC
*rplD* ^2^	GCCAACATTAAATTTATTCAATC	CTCGTAGTATTTAACAGCAC

^1^ Gene encoding L22 ribosomal protein, ^2^ Gene encoding L4 ribosomal protein.

## Data Availability

The data presented in this study are available on reasonable request from the corresponding author.
